# Long-Term Subjective and Objective Assessment of Smell and Taste in COVID-19

**DOI:** 10.3390/cells11050788

**Published:** 2022-02-24

**Authors:** Andrea Ciofalo, Carlo Cavaliere, Simonetta Masieri, Alessandra Di Chicco, Irene Fatuzzo, Federica Lo Re, Silvia Baroncelli, Elona Begvarfaj, Andrea Adduci, Ivano Mezzaroma, Claudio Maria Mastroianni, Marco de Vincentiis, Antonio Greco, Loris Zamai, Marco Artico

**Affiliations:** 1 Department of Sense Organs, Sapienza University, 00161 Rome, Italy; andrea.ciofalo@uniroma1.it (A.C.); alessandra.dichicco@uniroma1.it (A.D.C.); irene.fatuzzo@uniroma1.it (I.F.); federica.lore@uniroma1.it (F.L.R.); ebegvarfaj@yahoo.it (E.B.); andrea.adduci@uniroma1.it (A.A.); marco.devincentiis@uniroma1.it (M.d.V.); antonio.greco@uniroma1.it (A.G.); marco.artico@uniroma1.it (M.A.); 2 Department of Oral and Maxillofacial Sciences, Sapienza University, 00161 Rome, Italy; simonetta.masieri@uniroma1.it; 3 National Centre for Global Heath, Istituto Superiore di Sanità, 00161 Rome, Italy; silvia.baroncelli@iss.it; 4 Department of Translational and Precision Medicine, Sapienza University, 00185 Rome, Italy; ivano.mezzaroma@uniroma1.it; 5 Department of Public Health and Infectious Diseases, Sapienza University, 00161 Rome, Italy; claudio.mastroianni@uniroma1.it; 6 Department of Biomolecular Sciences, University of Urbino Carlo Bo, 61029 Urbino, Italy; loris.zamai@uniurb.it; 7 National Institute for Nuclear Physics (INFN), Gran Sasso National Laboratory (LNGS), 67100 L’Aquila, Italy

**Keywords:** COVID-19, smell, taste, B-SIT, taste strips

## Abstract

Among the first clinical symptoms of the SARS-CoV-2 infection is olfactory–gustatory deficit; this continues for weeks and, in some cases, can be persistent. We prospectively evaluated 162 patients affected by COVID-19 using a visual analogue scale (VAS) for nasal and olfactory–gustatory symptoms. Patients were checked after 7, 14, 21, 28, 90, and 180 days. A total of 118 patients (72.8%) reported an olfactory VAS < 7 at baseline (group B), and 44 (27.2%) reported anosmia (VAS ≥ 7) (group A) and underwent the Brief Smell Identification Test (B-SIT) and Burghart Taste Strips (BTS) to quantify the deficit objectively and repeated the tests to confirm the sense recovery. Group A patients showed B-SIT anosmia and hyposmia in 44.2% and 55.8% of cases, respectively. A total of 88.6% of group A patients reported ageusia with VAS ≥ 7, and BTS confirmed 81.8% of ageusia and 18.2% of hypogeusia. VAS smell recovery was recorded starting from 14 days, with normalization at 28 days. The 28-day B-SIT score showed normosmia in 90.6% of group A patients. The mean time for full recovery (VAS = 0) was shorter in group B (22.9 days) than in group A (31.9 days). Chemosensory deficit is frequently the first symptom in patients with COVID-19, and, in most cases, recovery occurs after four weeks.

## 1. Introduction

The SARS-CoV-2 infection, well known as COVID-19, has spread worldwide since February 2020, with pleomorphic symptomatic clinical manifestations. At the end of the first week of November 2021, the number of infected people globally amounted to about 250 million, while deaths totaled over 5 million [1].

The SARS-CoV-2 viral infection can cause upper airway congestion, hyposmia or anosmia, pharyngodynia, cough, fever, headache, general discomfort, diarrhea, and acute dyspnea to respiratory failure until death, mainly in fragile subjects. About 70% of infected patients are asymptomatic or paucisymptomatic; roughly 30% can have symptoms such as flu, and 25% of these patients need hospitalization, while about one-third of them may require treatment in an intensive care unit for respiratory distress [2]. It was observed that, among paucisymptomatic patients, the two main early symptoms are the loss of smell and taste with or without headache, fever, nasal obstruction, and rhinitis [3,4]. A report showed that almost half of the hospitalized patients presented anosmia and/or dysgeusia [5].

For this reason, smell and taste impairment is considered one of the first criteria for diagnosing COVID-19, although patients often show late taste deterioration. Various studies evaluated the evolution of taste and olfactory perception in patients affected by COVID-19, but only through questionnaires or telephone interviews due to difficulty approaching infected patients or administering validated olfactometric and gustometric tests in infectious disease clinics. As von Bartheld et al. observed, subjective methods such as questionnaires have lower sensitivity than objective tests on chemosensory loss [6]. The debate over the reliability and advantages of subjective or objective testing highlights the pros and cons of both methodologies. Faced often, but not always, with a lower prevalence of smell impairment observed in studies with subjective testing [7], Seok et al. reported that the self-assessment of the severity of the olfactory disorder correlates quite well with objective tests [8]. Finally, the already known limit of the most used objective tests such as the Burghart Sniffin’ Sticks and the University of Pennsylvania Smell Identification Test (UPSIT) relates to the set of odors tested that may not always be familiar to a country’s population different from the one where the test is validated [9].

This study aimed to evaluate, through the use of visual analogue scales (VASs), the prevalence of nasal symptoms and olfactory–gustatory deficit in COVID-19 patients, and to objectively measure, in the group of patients with a severe olfactory VAS score (VAS ≥ 7), the chemosensory deficit through the administration of validated smell and taste tests. All patients were monitored for up to 180 days from the COVID-19 diagnosis to estimate the time needed to recover the sensory function. Our study is the first single-center study performed with both subjective and objective olfactory and gustatory diagnostic methods on a group of 162 patients with 180 days of follow-up.

## 2. Materials and Methods

This is a monocentric prospective observational study. One hundred and sixty-two patients with a positive molecular swab for SARS-CoV-2 were enrolled after they were admitted to the infectious disease clinic of the Policlinico Umberto I Hospital in Rome. All patients had mild or moderate symptoms. Patient recruitment was between February and April 2021; the most represented COVID-19 variant in Italy was B.1.1.7 [10]. The study duration was six months for all patients, concluding the follow-up between August and October 2021.

The following inclusion criteria were considered: men or women aged 20 to 65; ability to understand and sign informed consent; first recent (no more than five days) positive swab for SARS-CoV-2 molecular test. The following exclusion criteria were considered: history of altered smell and taste before the pandemic of COVID-19; intubated patients; acute respiratory distress syndrome; nasal surgery within the previous six months; neurological diseases; head and neck oncological diseases.

Clinical data and chemosensory tests were collected in the patient’s room. We obtained informed consent, collected patient data, and filled in the questionnaire during the first medical examination (T0). The questionnaire consisted of physiologic anamnesis (age, sex, profession, weight, height, smoking status), family history, pathological anamnesis, ongoing therapies before and during COVID-19, upper airway symptom evaluation, and oxygen saturation (SpO_2_). All patients were asked to complete a VAS of olfactory-related symptoms such as sense of smell, sense of taste, nasal obstruction, rhinorrhea, postnasal drip, sneezing, and headache. The VAS is a quantitative measurement scale ranging from 0 (absence of symptoms/impairment) to 10 (extreme symptoms/impairment). All the patients who presented an olfactory VAS ≥ 7 (severe according to EPOS 2020 guidelines) [11] were assigned to group A; all these patients underwent the Brief Smell Identification Test (B-SIT) (Sensonics International, Haddon Heights, NJ, USA) and Burghart Taste Strips (BTS) (Burghart Messtechnik, Holm, Germany) to quantify the deficit objectively. All other patients with olfactory VAS < 7 were assigned to group B, including patients with VAS < 1 (normosmic). For our study, we chose to carry out the olfactory and gustatory tests, respectively, through the B-SIT, Italian version, and BTS as both methods are validated, undemanding for patients, and, above all, disposable.

The Brief Smell Identification Test [12,13] is a quick disposable screening test where the patient is asked to identify 12 odors contained in a microcapsule fixed on 12 strips of paper. This microcapsule is broken and scratched on with a pencil. The test is then administered to the patient, who must choose from four possible answers to identify the smell. A score under 9 points was considered hyposmia; a score less than or equal to 4 points was rated as anosmia [14]. The test was performed in 5 min, and both nasal nostrils were tested simultaneously.

Burghart Taste Strips [15] were used for the objective taste evaluation. This is another minimally invasive test, during which the patient tastes four different strips of paper at over-threshold concentration and has to report what type of taste has been perceived between sweet, salty, bitter, and acid. We considered normogeusia a score higher than 3, hypogeusia a score between 1 and 3, and ageusia a score less than 1. Patients were discharged from the hospital after a negative rhinopharigeal swab or, in case of repeated swab positivity after resolving the main symptoms (fever, asthenia), they were transferred to special non-hospital isolation facilities until swab negativization. To monitor symptoms, we conducted telephone interviews with all patients at 7 ± 2 days (T1), 14 ± 2 days (T2), 21 ± 2 days (T3), 28 ± 2 days (T4), 90 ± 7 days (T5), and 180 ± 7 days (T6). The B-SIT and BTS were repeated in all group A patients when the olfactory and taste VASs became less than a score of 7 to evaluate improvement objectively. The study duration was six months, from March 2021 to September 2021. Baseline B-SIT and BTS were performed after the morning round on the day of admission; the following tests were administered at the same hour during the follow-up visit.

The study was conducted following the Declaration of Helsinki and approved by the Ethics Committee of Policlinico Umberto I, Rome, Italy (protocol code 6531/1006). Informed consent from all patients was obtained during the first medical examination.

### Statistical Analysis

Data were analyzed using IBM SPSS Version 27.0 (IBM Corp, Somers, NY, USA). Results are expressed as the median and interquartile range for demographic analysis. The mean and 95% confidence interval (CI), percentage, and minimum/maximum were used for the results of testing. The continuous variable comparison between groups was performed using the Mann–Whitney U test. The Wilcoxon rank test was used for analyzing longitudinal changes through the follow-up period. Categorical variables were analyzed with chi-square or Fisher tests and advanced chi-square tests. Spearman’s correlation coefficient was used to evaluate correlations between quantitative variables. A value of *p* < 0.05 was considered statistically significant.

## 3. Results

One hundred and sixty-two patients (eighty females and eighty-two males, median age 57.0 years) were enrolled after a molecular swab confirmed COVID-19 infection. The Caucasian ethnicity was the most represented (97.7%). The epidemiological and clinical features of patients are reported in Table 1. Based on the olfactory VAS score, patients were dichotomized into group A (olfactory VAS score ≥ 7, *n* = 44) and group B (olfactory VAS score < 7, *n* = 118). No significant differences between groups were observed for age, BMI, and sex distribution. The prevalence of comorbidities was similar in the two groups.

All patients were hospitalized, suffering mild or moderate symptoms, without requiring ICU admission and/or supportive oxygen therapy. COVID-19-related symptoms appeared after a mean of 2.1 days from COVID-19 diagnosis, without differences between groups (*p* = 0.230) (Table 2). Fever was the most commonly reported complaint (64.2% of the patients), followed by respiratory symptoms and myalgia. Sp0_2_ levels were similar at study entry in the two groups, while group A patients reported more frequent asthenia and headache (*p* < 0.014 and 0.034, respectively) compared to group B patients.

### 3.1. Prevalence of Olfactory and Gustative Dysfunctions

At the first visit (T0), after a mean of 4.6 days from the COVID-19 diagnosis (CI: 4.0–5.0), the patients self-rated their olfactory abilities by a VAS, reporting the severity of the dysfunction on a scale of 0–10. Forty-four patients, 27.2% (CI: 20.2–34.1), reported anosmia with VAS ≥ 7 and were assigned to group A. The other 118 patients (72.8%) were allocated to group B, with 58% hyposmic (1 ≤ VAS < 7) and 14.8% normosmic (VAS < 1). The olfactory symptoms appeared 2.2 days after the COVID-19 diagnosis in 44% of the patients and even before the confirmed COVID-19 diagnosis in the other 56% (mean −1.4 days, CI: −1.9–−0.90). The severity of anosmia was correlated with other related nasal symptoms that were also evaluated by a VAS for nasal obstruction, rhinorrhea, postnasal drip, sneezes, and headache. Group A patients reported a higher prevalence of nasal obstruction (18.2% vs. 4.2%, *p* = 0.004) and headache (25% vs. 0.8%, *p* < 0.001). No differences were observed for rhinorrhea, postnasal drip, and sneezes. Generally, the 44 anosmic patients also showed higher VAS scores for all the olfactory-related nasal symptoms evaluated (Table 3). The objective olfactory evaluation was performed on all group A patients at baseline. The mean B-SIT score was 4.7 (CI: 4.40–4.98), confirming the olfactive dysfunction reported by patients in the subjective evaluation. Overall, all 44 patients had a B-SIT evaluation considered to be indicative of impaired smell; a total of 44.2% of them could not reach the score of 4, which implied a diagnosis of severe anosmia, and the other 55.8% of the patients had a score between 5 and 6.

**Figure 1:**
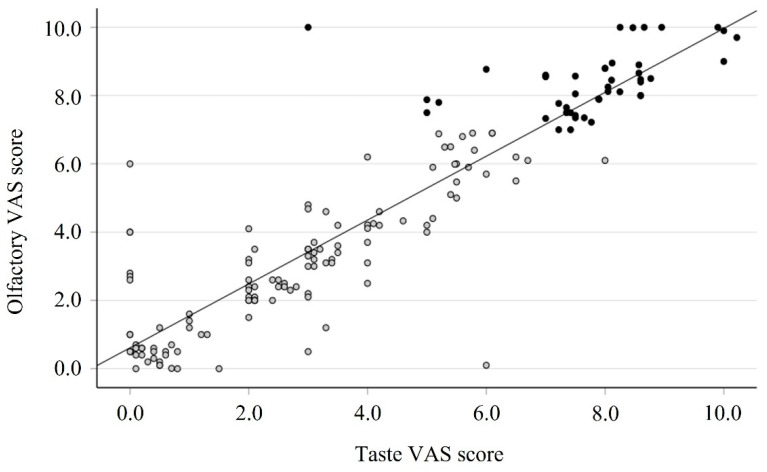
Correlation between olfactory and taste VAS scores in 162 patients after COVID-19 diagnosis (time 0). Black circle: group A; gray circle: group B; Spearman test: r = 0.906, *p* < 0.0001.

A total of 39 out of the 44 group A patients (88.6%) reported severe impairment in taste (ageusia, VAS ≥ 7); the mean of the taste VAS score at T0 was significantly different between group A and group B patients: 7.43 (CI: 7.03–7.83) vs. 2.47 (CI: 2.11–2.83), respectively, *p* < 0.001. For the objective taste evaluation, the Burghart Taste Strips test was used; at the study entry, 36 patients (81.8%) of the 44 group A patients failed to recognize any taste (score = 0), and the other 8 (18.2%) patients were able to taste only one sample, confirming a strong impairment of taste functions. The mean score for the BTS at baseline was 0.227 (CI: 0.07–0.39). The significant correlation confirmed the close relationship between the VAS score of smell and taste observed at baseline (r = 0.908, *p* < 0.0001, Figure 1).

### 3.2. Evolution of Olfactory and Gustative Dysfunctions

**Figure 2:**
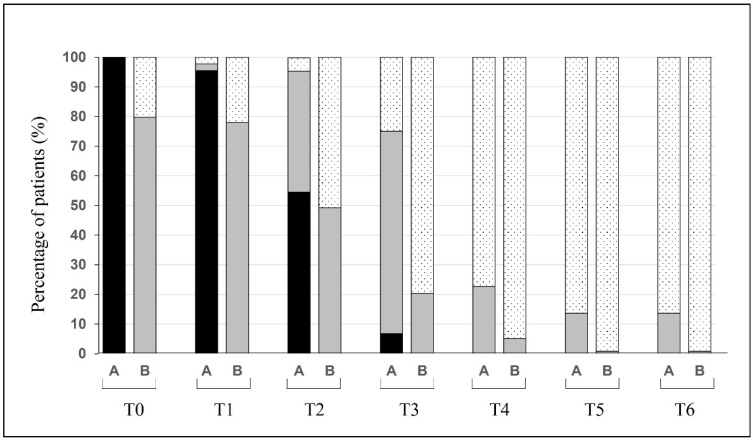
Changes over time of the percentage of patients (group A: anosmic; group B: hypo- and normosmic) suffering from different degrees of olfactory dysfunctions. Anosmia (black, VAS > 7), hyposmia (gray, 1 < VAS < 7), and normosmia (dotted, VAS < 1).

All 162 patients completed the follow-up period of the olfactory evaluation. In Figure 2, the recovery of smell dysfunction based on the VAS score is reported.

**Figure 3:**
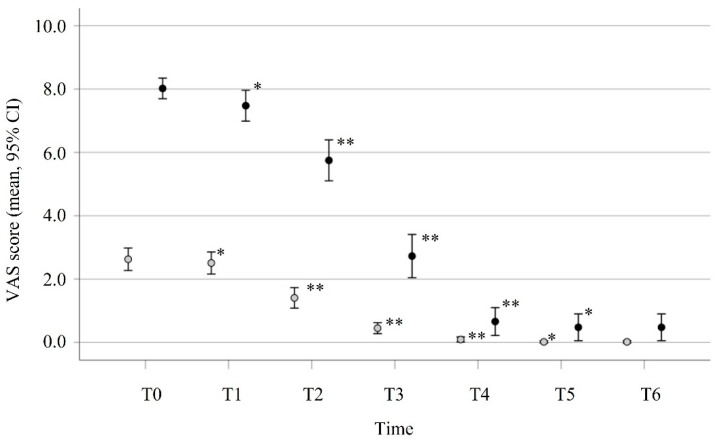
Evolution of olfactory dysfunctions in patients of group A (*n* = 44, VAS ≥ 7, black circle) and group B (*n* = 118, VAS < 7, gray circle). Each point represents the mean and 95% CI of the VAS score reported by patients during the study. Differences between time points were analyzed using the Wilcoxon test. * *p* < 0.05; ** *p* < 0.001.

In group A (Figure 2), the number of patients with a score ≥ 7 remained relatively stable during the first week (100% vs. 95.5%); then, from 28 days, the number of anosmic patients disappeared. At 180 days, still 13.6% of the patients reported a mild olfactory deficit. In group B (Figure 2), 24 (14.8%) patients were normosmic (VAS < 1) and did not experience any olfactory disturbance. The other patients with mild olfactory symptoms showed an evolution over time, and only one complained of an olfactory deficit at six months. The evolution in terms of the mean VAS score is reported in Figure 3.

The VAS score in group A decreased significantly from week 0 (mean score: 8.02, CI: 7.70–8.35) through week 4 (mean score: 0.66, CI: 0.22–1.09, *p* < 0.001). A similar trend was observed in the olfactory recovery of group B patients, where the mean of the VAS score, measured at week 0 (2.63, CI: 2.27–2.98), decreased significantly over 4 weeks (mean: 0.093, CI: 0.014–0.172). The mean time for full recovery (VAS = 0) was significantly different between groups: 22.9 days (CI: 19.4, 26.3) in group B, and 31.9 days (CI: 25.1–38.8) in group A (*p* = 0.010). According to the olfactory VAS score, one patient from group B (VAS score = 2) and six patients (13.6%) from group A (mean VAS score 3.5) still had olfactory impairment after 180 days from infection.

**Figure 4:**
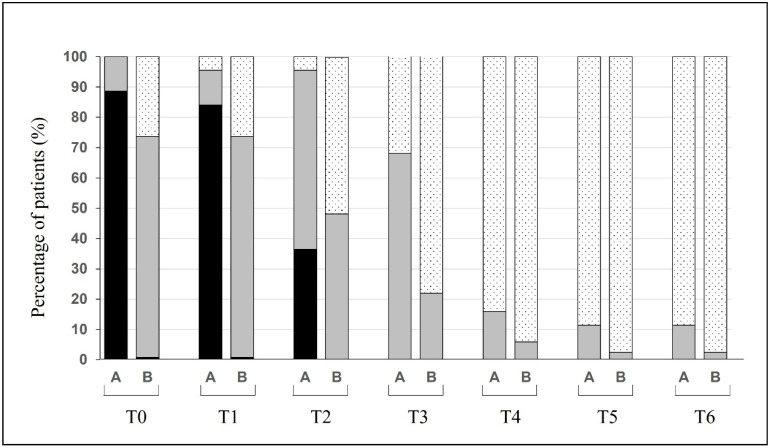
Changes over time of the percentage of patients (group A: anosmic; group B: hypo- and normosmic) suffering from different degrees of taste dysfunctions and their evolution over time. A: group A; B: group B. Ageusia (black, VAS ≥ 7), hypoageusia (gray, 1 ≤ VAS < 7), and normosmia (dotted, VAS < 1).

The recovery of olfactory functions was paralleled by an improvement in taste measured by the VAS. During the first two weeks from COVID-19 diagnosis, the number of group A patients with severe impairment of taste functions (VAS ≥ 7, ageusia) remained quite stable (39 and 37 patients, respectively). A progressive recovery in taste was observed on day 21, when 36.4% of the patients still reported a VAS ≥ 7, and from day 28, all patients reported only mild taste impairment (30, 68.2%) or none (Figure 4).

### 3.3. Objective Olfactory and Gustative Dysfunctions

**Figure 5:**
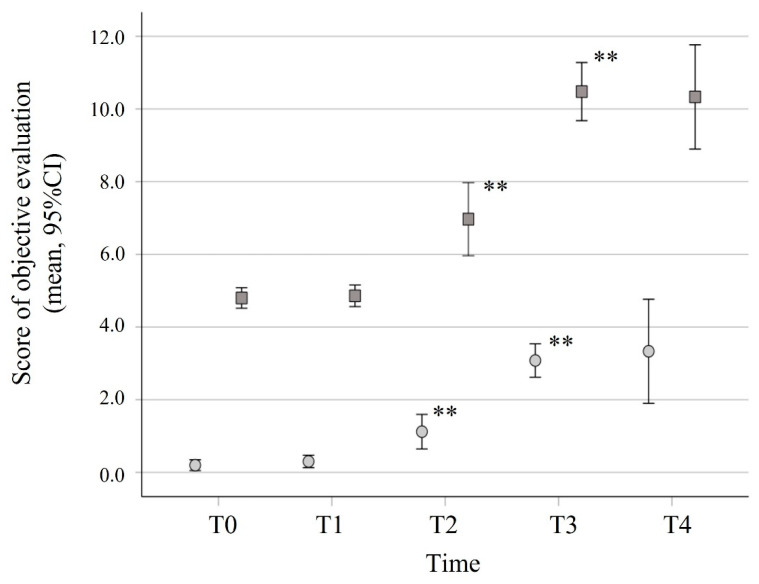
Recovery of olfactory and taste functions measured by objective evaluation tests. B-SIT (square) and BTS (circle) scores were obtained during the first 4 weeks from COVID-19 diagnosis in all group A patients. Differences between time points were analyzed using the Wilcoxon test. ** *p* < 0.001.

The olfactory and gustative dysfunctions were objectively evaluated in group A patients using the B-SIT and the BTS at different time points (Figure 5). The improvement in olfactory functions measured by the B-SIT was observed at the different time points (T2, T3, and T4), and the score increased significantly until a mean score of 10.7 (CI: 9.8–11.5) on day 28. The BTS test was used for the objective taste evaluation. At T2, most group A patients (54.5%) still had ageusia. At T4, 81.4% of the patients had a BTS score = 4, indicating full recovery (mean = 3.59, CI: 3.37–3.81). The timing and the degree of smell and taste recovery were concomitant in all patients, as indicated by the significant correlations between B-SIT and BTS scores at different time points (*p* < 0.001, Figure 5).

## 4. Discussion

The SARS-CoV-2 outbreak has infected over 250 million people worldwide. Studies from various countries reported that the symptomatic disease occurs with fever, cough, dyspnea, fatigue, pharyngodynia, rhinorrhea, and headache. Another distinctive aspect in these patients, from the first days of illness, is the loss of the sense of smell often associated with impaired taste.

Since March 2020, numerous studies have studied the impairment of smell and taste in COVID-19; these were conducted on a variable number of patients with different endpoints. Jafar et al. found a total of 44 papers and selected 14 papers performed on mild to moderate symptomatic patients; sample sizes ranged from 7 to 300 patients, with a few objective olfactory assessment tools such as the UPSIT, the Burghart Sniffin’ Sticks, the B-SIT, the Connecticut Chemosensory Clinical Research Center (CCCRC) tests, and the self-administered psychophysical evaluation-ethyl alcohol olfactory thresholds [16]. The percentage of anosmia between selected papers ranged from 0% to 47%, hyposmia ranged from 25.4% to 100%, and normosmia ranged from 0% to 36.7%. In 26 papers with subjective olfactory measures (VAS or questionnaire), the prevalence of anosmia was from 3% to 83%, that of hyposmia was from 5.1% to 67%, and that of normosmia was from 0% to 89.3%. Finally, four studies used objective and subjective olfactory measures, and anosmia was found to range from 10% to 72.7%, hyposmia from 18.4% to 53.3%, and normosmia from 0% to 49.4%.

In our study, we wanted to observe the prevalence of smell and taste impairment in COVID-19 patients and evaluate their recovery times; the first tool we used to define the alteration of smell and taste was a VAS. The 162 patients we studied showed anosmia in 27.2% (44 patients), hyposmia in 58% (94 patients), and normosmia in 14.8% (24 patients) of cases at baseline. The 44 patients with reported anosmia (VAS ≥ 7, group A) underwent the objective B-SIT that confirmed anosmia in 44.2% of cases and hyposmia in 55.8% of cases, with no normosmic patients. These values are different from the other two papers that used the B-SIT. Ugurlu, in 42 patients, reported 16.7% of anosmia, 83.4% of hyposmia, and 0% of normosmia [17], while Bertlich, in 23 patients, reported, respectively, 72.7%, 27.3%, and 0% [18]. The difference in the percentages compared to these two works is probably due to different sample sizes and the fact that, in our study, we performed the B-SIT only on patients already selected by an olfaction VAS score ≥ 7, demonstrating that there may be a discrepancy between the patients’ subjective perception as observed by other authors [19]. In the other two studies that used the B-SIT, there were differences in the percentages of anosmic patients, probably due to the difference in the average age compared to our work, which was 57 ± 6. Specifically, the mean age in Bertlich’s work was 59 years (±16.6), with 72.7% of anosmia, while in Ugurlu’s work, the mean age was 41.2 years ± 14.6, with 16% of anosmia. Furthermore, the other studies had a different male/female ratio than ours. The physiological difference in olfactory perception due to sex, which is better in females, and the decrease in smell based on aging could make the olfactory perception different at the baseline and after viral infection [20,21].

The olfactory mucosa consists of a neuro-olfactory epithelium, which is formed by five types of cellular elements: olfactory neurons, bipolar cells with cilia, on which the olfactory receptors are located; sustentacular cells, which protect neurons and support their activity and metabolism by releasing glucose into the mucus; microvillar cells, which are thought to play a role in modulating the olfactory signal; basal cells, which represent the stem cells able to regenerate the olfactory epithelium; and Bowman’s glands that produce mucus which helps odorous molecules bind to olfactory receptors [22,23].

The inflammation of the olfactory epithelium in COVID-19 seems to be supported by the release of cytokines such as IL-1, IL-6, IL-12, IL-15, and TNF-α, and by activating lymphocytes and macrophages, initiating the so-called “cytokine storm” [24]. IL-6, in particular, activates the pathway that leads to cellular apoptosis of the olfactory epithelium; it has been shown that a decrease in IL-6 levels favors the recovery of smell after post-COVID-19 anosmia [25].

Inflammation also compromises olfactory pathways as leukocyte infiltrates have been found in olfactory bulbs of patients who have died from COVID-19 [26]. The passage of the virus towards the olfactory mucosa might occur by passive diffusion and axonal transport through the cribriform plate of the ethmoid bone towards the central nervous system [27,28]. However, other studies are needed to understand this mechanism of diffusion better. In fact, Khan argued that no infection evidence of olfactory neurons has been found and that these are suffering without being infected [29]. However, Morbini et al. demonstrated the finding of SARS-CoV-19 viral particles in the bulb in patients who died from this infection [28]. These particles were associated with diffuse infiltration of CD163-positive macrophages and cytotoxic T lymphocytes as a macrophage activation marker induced by the proinflammatory cytokine storm in systemic inflammatory disorders. These findings suggest that passive diffusion and axonal transport through the olfactory complex may be a major route of SARS-CoV-2 entry into the central nervous system, as it was previously shown in animal studies with a human coronavirus strain [30]. Interestingly, in a hamster model of SARS-CoV-2 infection, nasal instillation of SARS-CoV-2 seemed to be able to induce infection of sustentacular cells but not of olfactory neurons, and a massive immune cell infiltration in the olfactory epithelium was also observed [31]. Therefore, SARS-CoV-2-induced local immune responses may indirectly play a role in damaging olfactory receptor neurons in COVID-19. Indeed, elevated levels of proinflammatory cytokines such as TNF-α [24] and cytotoxic/helper T lymphocyte infiltration [26] were observed in the olfactory epithelium of patients with COVID-19, raising the possibility that an indirect contribution by immune cell responses against the olfactory epithelium may participate in anosmia in COVID-19 patients. However, oolfactory sensory neurons lost most of their cilia and therefore their function, suggesting an indirect deterioration of olfactory neurons after the infection of the olfactory epithelium; nevertheless, no evidence of SARS-CoV-2 detection in or transfer from sustentacular cells to olfactory receptor neurons or axonal transport of SARS-CoV-2 to the brain has been demonstrated in the hamster infection model [31]. This is in line with recent reviews of the topic [32,33]; however, the ability of SARS-CoV-2 to enter the brain upon intranasal infection remains controversial [34].

On the other hand, since part of the non-infected cells was desquamated in response to SARS-CoV-2 infection, Bilinska and colleagues proposed the hypothesis that the infection and consequent impairment of sustentacular cell function may lead to olfactory neuronal death and dysosmia in COVID-19 patients [32]. Indeed, dysosmia is often reported in mild cases, suggesting the existence of a sustentacular-dependent mechanism activating immune responses [32]. However, a viral infection of sustentacular cells has never been clearly correlated with the alteration of smell perception [32]. While neuroimaging revealed localized inflammation in the intracranial olfactory structures in humans, to date, histopathological, ultrastructural, and molecular evidence does not suggest that SARS-CoV-2 is an obligate neuropathogen [35].

Our study found 85.2% of the patients with subjective olfactory disorder between anosmic (27.2%) and hyposmic (58%). The B-SIT further refined the staging of olfactory impairment by discriminating between patients deemed anosmic, according to the EPOS 2020 criteria (olfactory VAS ≥ 7), and those with severe anosmia (44.7%); furthermore, objective testing confirmed that 100% of the patients in group B were anosmic according to the B-SIT scoring system. This suggests that the observation by Seok et al. on a good correlation between subjective and objective testing may be correct; in fact, the authors observed that olfactory test scores significantly correlate with self-rated scales but noted a discrepancy between olfactory test results and the severity described by patients [8].

Dysgeusia or complete taste loss can be associated with COVID-19; often, patients report the loss of taste based on the inability to recognize the gustatory flavors rather than the perception of primary tastes such as sweet, bitter, salty, and sour. This condition is commonly related to the critical postnasal olfactory component, stimulated by the airflow between the mouth and nasopharynx created by chewing, which cannot fulfill the appreciation and recognition of food flavors due to the concomitant total or partial smell deficit [36,37]. Pathological mechanisms that determine dysgeusia in subjects affected by COVID-19 can vary. Among the hypotheses, it is thought that, as for the olfactory mucosa, the expression of ACE-2 receptors in the oral mucosa, ascertained in the taste buds, can favor the infection and therefore induce a “cytokine storm” due to TNF-α, IL-6, and IFN-γ overexpression [38]. Another mechanism that can cause the taste impairment concerns the high expression of ACE-2 receptors and Transmembrane Serine Protease 2 (TMPRSS2) in the salivary glands, which can lead to a quantitative and qualitative deficit of salivary production [39,40,41]. Some studies observed that COVID-19 compromises the cranial nerves (VII, IX, X pairs), altering taste perception; in particular, the VII pair of nerves can be reached by the virus through the nasopharynx and the Eustachian tube to the middle ear involving the chorda tympani, which set taste sensitivity [42].

Evaluating symptom onset and the diagnosis is essential to confirm that the chemosensory deficit is a sign of alarm. The literature reports a wide difference in diagnosis timing; patients enrolled in this study presented with mild–moderate symptoms that appeared, on average, 2.1 days from the diagnosis of COVID-19 infection and were evaluated, on average, over the following 4.9 days. Similar to our experience, Moein et al. evaluated patients after 4 days [43], Iannuzzi et al. after around 20 days [44], and Vaira et al. after 2–35 days [45].

Taste and smell deficits are often complained of by patients simultaneously after COVID-19 infection. In a meta-analysis of 104 studies with 38,198 patients, von Bartheld noted that the prevalence of olfactory deficit was 43%, and that of gustatory deficit was 44.6% [6]. Regarding the evaluation of taste, the authors used various, often not validated, methods, thus obtaining questionable answers [46]. Our study used a validated gustometry test, the Burghart Taste Strips, and found a close correlation between the reported occurrence of olfactory and gustatory deficits and the objective response.

We observed that the recovery of the two senses occurred in the great majority of cases in parallel during the four weeks following the first evaluation. According to the subjective survey, after 28 days, 90.1% of the patients totally recovered their sense of smell, and the remaining 9.9% still complained of hyposmia; we think they may still have a peripheral or central inflammatory state unknown to us that prevents them from optimal subjective perception. A total of 95.7% of the patients reported subjective normalization after the 180-day follow-up. The B-SIT showed a mean score of 10.7, with 38 (86.4%) normosmic patients, and 6 (13.6%) still hyposmic after four weeks. The recovery of taste showed a similar trend, with normalization in 81.4% of the patients after 28 days. These results are similar to those reported in other papers [18,47,48,49].

The recovery of smell and taste in patients who do not undergo spontaneous recovery remains a problem in the therapeutic management of chemosensory deficits from viruses and, therefore, also from COVID-19. Consensus on the treatment of postinfectious olfactory dysfunction underlines how olfactory training and steroids are supported by good scientific evidence for the treatment of postinfectious anosmia [50]. In particular, the combination of oral and topical steroids would seem to improve the outcome compared to topical steroids alone [51].

Some therapeutic schemes have been proposed based on the administration of systemic and local corticosteroids to counteract the inflammation, but this exposes patients to side effects and the risk of worsening the systemic damage caused by the virus [52]. However, in a recent study, Le Bon [53] suggested that the combined effect of 10-day oral corticosteroid therapy and olfactory training, i.e., stimulation of the sense of smell with four substances (rose, lemon, eucalyptus, and cloves) twice a day for three months, improved the sense of smell in patients with a persistent olfactory deficit [54]. On the other hand, Abdelalim et at. performed a prospective randomized controlled trial observing that the use of a mometasone furoate nasal spray offered no superiority compared to olfactory training in terms of recovery rates or duration of smell impairment, while training alone seemed to be advisable for COVID-19 patients [55].

## 5. Conclusions

Chemosensory deficit affects a large proportion of COVID-19 patients, often representing the onset symptom of the infection. There are differences in the evaluation of olfactory–gustatory symptoms between subjective and objective methods using psychophysical tests. The most significant percentage of patients regain the sense of smell and taste after about four weeks, and only in a few does the deficit last over six months. There is a difference between subjective and objective testing; the literature shows this difference in terms of prevalence between the two evaluation methods, even if the results seem to be correlated with each other. In everyday clinical practice, objective tests lead to an increase in the costs and time of patient evaluation that the B-SIT can mitigate as it is reasonably quick, requiring only five minutes, safe, in the case of infected patients, and less expensive than other methods.

Concerning recovery times, most patients recover between 22 and 31 days depending on the initial grade of sense impairment; about 10% of severe patients need a longer time to get better, perhaps due to central involvement. Further studies are needed to better evaluate the lack of chemosensory recovery, due to central or peripheral mechanisms, beyond six months since infection.

## Figures and Tables

**Table 1:** Epidemiological and clinical features of 162 patients at the diagnosis of COVID-19. Group B: olfactory VAS score < 7; group A: olfactory VAS score ≥ 7. Values are reported as the median and IQR or percentage.

	Total Population	Group A	Group B	*p* Values
No. of patients	162	44	118	
Age (years)	57.0 (48.8–63.0)	57.0 (51.0–64.0)	56.0 (46.8–63.0)	0.239
Sex (female)	80 (49.4%)	27 (61.4%)	55 (46.6%)	0.113
BMI	26.9 (24.8–29.5)	27.1 (25.1–30.1)	26.7 (24.7–29.3)	0.292
Current smoker (*n*, %)	33 (20.4%)	10 (22.7%)	23 (19.5%)	0.665
Infectious diseases (*n*, %)	0	0	0	-
Autoimmune diseases (*n*, %)	22 (13.6%)	12 (27.3%)	10 (8.5%)	0.004
Hypertension (*n*, %)	45 (27.8%)	16 (36.4%)	29 (24.6%)	0.168
Respiratory insufficiency (*n*, %)	17 (10.5%)	7 (15.9%)	10 (8.5%)	0.246
Heart problems (*n*, %)	61 (37.7)	18 (40.9%)	43 (36.4%)	0.716
Kidney insufficiency (*n*, %)	6 (3.7%)	4 (9.1%)	2 (1.7%)	0.047
Allergies (*n*, %)	10 (6.2%)	1 (2.3%)	9 (7.6%)	0.108
Comorbidities (*n*, %)	89 (54.9%)	28 (63.6%)	61 (51.7%)	0.215

**Table 2:** Frequencies of symptoms during the acute phase of COVID-19 infection in patients. Values are expressed as the median and IQR or percentage. * *p* < 0.05.

	Total Population	Group A	Group B	*p* Values
*n*	162	44	118	
Symptomatic (*n*, %)	149 (92.0)	39 (88.6)	110 (93.2)	0.547
Onset of COVID-19 symptoms (days)	2.1 (1.9–2.4)	2.5 (1.8–3.2)	2.0 (1.7–2.3)	0.230
SpO_2_ (%)	95 (93.8–97)	95 (94–97)	95 (93–97)	0.447
Fever (*n*, %)	104 (64.2)	27 (61.4)	77 (65.3)	0.713
Asthenia (*n*, %)	77 (47.5)	28 (63.6)	49 (41.5)	0.014 *
Respiratory symptoms (*n*, %)	55 (34.0)	16 (36.4)	39 (33.1)	0.712
Myalgia (*n*, %)	33 (20.4	8 (18.2)	25 (21.2)	0.827
Gastrointestinal symptoms (*n*, %)	13 (8:0)	1 (2.3)	12 (10.2)	0.189
Headache (*n*, %)	27 (16.7)	12 (27.3)	15 (12.7)	0.034 *

**Table 3:** Prevalence and VAS score of olfactory dysfunctions in patients of group A and group B at time 0. Values are expressed as a percentage and mean and 95% CI. * *p* < 0.05.

	Group A	Group B	*p* Values
Anosmia (*n*, %)	44 (27.2%)	118 (72.8%)	<0.001 *
VAS score	8.02 (7.70–8.35)	2.63 (2.27–2.98)	
Ageusia (*n*, %)	39 (88.6%)	1 (0.8%)	<0.001 *
VAS score	7.43 (7.03–7.83)	2.47 (2.11–2.83)	
Nasal obstruction (*n*, %)	8 (18.2%)	5 (4.2%)	0.007 *
VAS score	4.91 (4.19–5.63)	2.32 (1.94–2.70)	
Rhinorrea (*n*, %)	0 (0%)	3 (2.5%)	0.286
VAS score	3.86 (3.35–4.38)	1.77 (1.42–2.12)	
Postnasal drip (*n*, %)	0 (0%)	4 (3.4%)	0.216
VAS score	3.50 (3.00–4.00)	1.57 (1.11–1.92)	
Sneezes (*n*, %)	1 (2.3%)	1 (0.8%)	0.465
VAS score	3.18 (2.61–3.75)	1.72 (1.40–2.04)	
Headache (*n*, %)	11 (25%)	1 (0.8%)	<0.001 *
VAS score	4.66 (3.86–5.45)	2.04 (1.67–2.41)	

## Data Availability

The data presented in this study are available on request from the corresponding author. The data are not publicly available due to ethical reason.
